# The Endocannabinoid System in Glial Cells and Their Profitable Interactions to Treat Epilepsy: Evidence from Animal Models

**DOI:** 10.3390/ijms222413231

**Published:** 2021-12-08

**Authors:** Jon Egaña-Huguet, Edgar Soria-Gómez, Pedro Grandes

**Affiliations:** 1 Department of Neurosciences, Faculty of Medicine and Nursing, University of the Basque Country UPV/EHU, 48940 Leioa, Spain; jon.egana@ehu.eus; 2 Achucarro Basque Center for Neuroscience, Science Park of the UPV/EHU, 48940 Leioa, Spain; 3 IKERBASQUE, Basque Foundation for Science, 48009 Bilbao, Spain; 4 Division of Medical Sciences, University of Victoria, Victoria, BC 3800, Canada

**Keywords:** endocannabinoid system, glial cells, epilepsy, neuroinflammation

## Abstract

Epilepsy is one of the most common neurological conditions. Yearly, five million people are diagnosed with epileptic-related disorders. The neuroprotective and therapeutic effect of (endo)cannabinoid compounds has been extensively investigated in several models of epilepsy. Therefore, the study of specific cell-type-dependent mechanisms underlying cannabinoid effects is crucial to understanding epileptic disorders. It is estimated that about 100 billion neurons and a roughly equal number of glial cells co-exist in the human brain. The glial population is in charge of neuronal viability, and therefore, their participation in brain pathophysiology is crucial. Furthermore, glial malfunctioning occurs in a wide range of neurological disorders. However, little is known about the impact of the endocannabinoid system (ECS) regulation over glial cells, even less in pathological conditions such as epilepsy. In this review, we aim to compile the existing knowledge on the role of the ECS in different cell types, with a particular emphasis on glial cells and their impact on epilepsy. Thus, we propose that glial cells could be a novel target for cannabinoid agents for treating the etiology of epilepsy and managing seizure-like disorders.

## 1. Epilepsy and Neuroinflammation

Epilepsy is a prevalent disease in our society, being one of the most common neurological conditions affecting 1–3% of people worldwide [1]. This condition has a high impact on the patient’s life, leading to long-term cognitive impairment. Around 70% of the patients present verbal or episodic memory decline, decreased attention, executive functions or psychomotor issues, and depression [2]. The development of epilepsy involves a variety of molecular modifications leading to aberrant synaptic reorganization. Such changes result in abnormal synchronized neuronal firing and uncontrolled excitability. These events produce spontaneous recurrent episodes of symptoms, known as seizures, which entail excitotoxicity and cell death [3,4]. The affected brain regions, mechanisms of action, and pathological manifestations vary depending on the type of seizure-related disorders. For example, idiopathic epilepsies are a result of genetic alterations. On the other hand, acquired epilepsies are caused by traumatic brain injuries or stroke, where epileptic symptoms can arise time before the onset of spontaneous recurrent seizures [5]. The time spent between the first seizure episode and the chronic disease is known as the latent period or epileptogenesis. There, brain alterations occur to end up on an imbalance between excitatory and inhibitory neurotransmission, affecting normal brain functions [6].

Over the last few decades, different studies have shown that inflammation processes driven by glial cell activation and the release of pro-inflammatory cytokines contribute to neuronal damage and, therefore, to epileptogenesis, underlying epilepsy-related disorders [7,8,9]. Furthermore, clinical and experimental research points out that those persistent levels of cytokines in the brain can act as facilitators of cell death. In addition, these molecules could decrease seizure susceptibility or even augment neuronal excitability during the progression of the disease [10,11,12].

The role of the endocannabinoid system (ECS) in controlling neural network excitability has been the focus of intense pharmacological interest, developing therapies based on the use of cannabinoid compounds for the control of seizures [13,14,15,16]. Moreover, ECS protects the brain cells by controlling glutamatergic neuronal excitability through the type 1 cannabinoid receptor (CB_1_R) activity, the main cannabinoid receptor present in the brain [17,18,19,20]. Not only CB_1_R is deeply investigated in the treatment of epilepsy, but also other receptors that also interact with endogenous or exogenous cannabinoids have been postulated as potential targets in the control of seizures. Indeed, in the last ten years, the number of articles published on PubMed in relation to “cannabis and epilepsy” has grown more than 10-fold [21]. Therefore, studying the molecular mechanisms triggered by cannabinoid action could bring novel insights into the understanding of epileptic disorders and related pathophysiological processes.

## 2. Etiology and Treatment

Nowadays, one of the biggest concerns with epilepsy is the high incidence of resistance to current treatments with antiepileptic drugs (AEDs) [16]. In the last decade, different approaches have attempted to prevent seizures and to control them. These treatments, some obtained from pre-clinical studies, ranging from pharmacological therapies based on new compounds such as resveratrol or vitamin E [22,23]; cell therapies rooted on stem cells, interneuron precursor transplants [24,25,26] or reprograming reactive glia into interneurons [27], and gene therapies such as neuropeptide Y transfection [28,29] to clinical studies in humans, such as modern surgery with laser-induced thermal therapy (LITT); magnetic resonance-guided LITT (MRgLITT) [30,31]; brain stimulation [32,33], or even controlling seizures with diet such as a ketogenic diet or other regimens [34]. Since most AEDs available in the market are only preventing and treating the symptoms instead of acting on the molecular mechanisms, they produce numerous negative side effects that limit their therapeutic use [35,36,37]. Some treatments increase GABAa receptor activation in the brain, therefore increasing inhibitory tone. Other approaches block sodium or calcium channels to prevent cell depolarization and signal transduction [20]. Hippocampal sclerosis is the most common pathological feature of temporal lobe epilepsy (TLE) in humans that is associated with a significant loss of CA1 pyramidal neurons and prominent reactive astrogliosis [11]. However, the cellular mechanisms promoting abnormal remodeling of neuronal networks in epilepsy are not fully described [38,39]. Consequently, it is crucial to understand such mechanisms in order to elucidate the pathophysiology of the epilepsies, intending to generate novel therapeutic targets useful in seizure control [40].

Human studies have shown the relationship between inflammation, immunity, and epileptic susceptibility in TLE [41,42,43]. As mentioned before, neuroinflammatory events can potentiate neuronal hyperexcitability and the spread and recurrence of seizures [10,44,45,46]. Recent reports have demonstrated the involvement of oxidative stress and reactive oxygen species (ROS) in the development of post-traumatic epilepsies. Furthermore, oxidative processes potentiate the effects produced by neuroinflammation itself and outline the importance of controlling such events in the prevention and control of seizures [47]. Therefore, manipulating inflammatory signaling cascades also constitutes a complementary therapeutic approach for treating epilepsies [48]. For instance, some of the pro-inflammatory molecules that have been linked to epilepsy, and thus could contribute to the progression of seizures and epileptogenesis, are cyclooxygenase-2 (COX-2), prostaglandin receptor EP_2_ (PGEP_2_), interleukin 1β and 6 (IL-1β, IL-6), transforming growth factor β (TGFβ), or tumor necrosis factor α (TNFα), among others [49,50].

Up to date, most research on epilepsy has been devoted to the study of neuronal mechanisms. However, in the last decade, new reports have focused on non-neuronal cell contributions to the development of the disease [8,51,52,53,54,55], namely glial cells that sustain neurons by different mechanisms [56]. A wide range of glial cell-driven modifications occurs during epileptogenesis, including neuroinflammation [57] and changes in brain architecture [8,58,59]. In this sense, astrocytes and microglia deserve special attention since their intrinsic roles in tissue maintenance and repair might be critical in epilepsy development. Thus, future research should consider these cells as essential mediators in the disease, highlighting a new therapeutic target to treat seizure disorders [60,61]. Oligodendrocyte dysfunction also may alter normal brain functioning in several disorders since oligodendrocyte-driven myelination affects axon conduction velocity and signal propagation timing, having a direct impact on neuronal and glial communication. [56]. Nevertheless, although the oligodendroglial participation in epilepsy is so far limited, these glial cells could represent new targets for cannabinoid-based treatments of other brain disorders [62].

## 3. ECS on Epilepsy

The plant Cannabis sativa has been used as an antiepileptic drug [63], although the great variability in patients’ response and the psychoactive effects are limiting factors in its application. Although some plant-derived cannabinoids are being employed together with AEDs in seizure control, possible long-term effects that could reduce the antiepileptic action or even aggravate the seizures are the main limitations for the clinical use of cannabis [15,16,64,65]. Several reviews have compiled the effects of different cannabinoid compounds on various models of seizures in rodents [20,66,67,68,69] and in patients with different epileptic disorders [70,71].

CB_1_R activation has been described to be protective against excitotoxicity [17,18] and brain injury [72]. To this extend, neuronal CB_1_R expression is altered and reorganized in several models of TLE [73,74,75,76,77,78]. Apart from being located in neurons (principal and interneurons) [79] and neuronal subcellular compartments and organelles such as mitochondria [80,81,82,83,84,85,86], CB_1_Rs are localized in glial cells such as astrocytes and oligodendrocytes although to a much lower extent [85,86,87,88], where it modulates synaptic transmission and plasticity [89,90,91]. Interestingly, CB_1_Rs are also in astrocytic mitochondria, where they regulate astroglial glucose metabolism and social interactions [84,92,93]. Moreover, relatively low CB_1_R mRNA has been detected in cortical microglia of newborn rats [87,94,95,96]. On the other hand, CB_2_R is mostly localized in microglia and macrophages [97,98,99], and some reports have shown CB_2_R in neurons [100,101,102]. Although CB_2_R expression in the CNS is very restricted compared to CB_1_R, its upregulation in response to different harmful stimuli [102,103] plays active functions in neurological processes, such as nociception, drug addiction, or neuroinflammation [104,105]. Likewise, several studies show that CB_2_R participates in neuroprotection in different models of epilepsy and excitotoxicity [106,107,108,109].

In addition, endogenous (eCBs) and exogenous cannabinoids interact with other receptors [110,111,112] that could directly influence the development and progression of epilepsy. Among these receptors, G-protein coupled receptor 55 (GPR55) [113], nuclear peroxisome proliferator-activated receptor alpha (PPARα) [114,115], transient receptor potential vanilloid type 1 (TRPV1) [114,116,117], or abnormal cannabidiol receptor (abn-CBDR) have been postulated and are currently recognized as cannabinoid-receptors [118].

### Review Statement

Cannabinoid compounds elicit anti-inflammatory and neuroprotective responses against brain insults [119,120]. Glial malfunctioning can cause a hypersynchronous-like state and hyperexcitability, together with maintained inflammatory alterations that can promote epileptogenesis and provoke seizures [8]. The activation of CB_1_R and CB_2_R in glial cells exerts neuroprotection by increasing anti-inflammatory and decreasing pro-inflammatory cytokines, among others [121,122,123]. In this work, we review the state of the art of cannabinoid effects on glial cells and their impact on neuroinflammation and epileptic disorders.

## 4. Microglia and ECS in Epilepsy

### 4.1. Microglia

Microglial cells respond rapidly to pathological events occurring in the surrounding that lead to their activation and changes in their normal functioning. Thus, these glial cells express pattern recognition receptors (PRR), viral receptors, or toll-like receptors (TLR) on their surface [124] that allow them to detect signals from other cells and environmental states in order to sense very subtle changes induced by lifestyle-related factors (cognitive stimulation, exercise, diet, stress) [125,126]. Microglial activation includes migration, morphological changes, and proliferation, depending on the polarization or phenotype triggered. Hence, microglia states can shift from resting M0 to activated pro-inflammatory M1 or anti-inflammatory M2 [127]. Furthermore, these modifications can promote interactions with neurons and astrocytes, causing a general neuroinflammation and brain homeostasis alteration [128,129,130,131,132,133].

On the other hand, microglial cells are able to release several molecules involved in neuroprotection [134,135,136]. Thereby, microglia could release endocannabinoids, regulating synaptic transmission and plasticity through different mechanisms [133,137,138]. For instance, anandamide released by primary microglia activates CB_1_Rs at GABAergic interneurons reducing inhibitory synaptic transmission [133,137]. Therefore, the ECS in microglia could be a promising target in the control of brain excitability.

### 4.2. Microglia and Inflammation in Epilepsy

The participation of microglia in modulating neuronal networks under seizure conditions is not fully understood [52]. Thus, some studies have linked microglia to the inflammatory constituent of the disease. In contrast, others have reported a worsening of the disease’s progression after the genetic ablation of microglial cells, having a negative impact on both behavior and brain activity as well as on the neurodegeneration triggered after the status epilepticus [139]. In animal models, either chemoconvulsants or electrical stimulation induce seizures and status epilepticus, eliciting brain modifications that lead to neuronal hyperexcitability [52]. Those changes are accompanied by microglial activation in specific brain areas and the release of inflammatory mediators [140,141], which could favor or delay the onset of the disease [52,57,139]. Thus, it is proposed that microglial activity supports epileptogenic processes in the brain, contributing to disease development.

### 4.3. Microglia and ECS in Epilepsy

#### 4.3.1. Cannabinoid Receptors

There are pieces of evidence indicating that CB_1_ and CB_2_ receptors are expressed in microglia depending on microglial conditions [87]. Thus, microglia hardly expressed (if any) CB_1_R and CB_2_R at resting conditions. In fact, specific CB_1_R antibodies were unable to detect CB_1_R in microglial cells of the healthy brain. However, CB_1_R was observed in cultured microglia of several species [95,104,121,142]. As to CB_2_R, only some mRNA has been detected in healthy brain, suggesting that this receptor is not expressed much in quiescent microglia [97,143,144]. In the brain, CB_2_R is expressed in activated microglia in certain conditions. For instance, neuropathic pain increases CB_2_R in microglia of the spinal cord that is not seen in chronic inflammatory pain [145]. There is also an increase in microglial CB_2_R in inflammation [146] and in activated microglia in brain tissue of patients with Alzheimer’s disease or multiple sclerosis, mostly at the lesion sites [147,148], in the vicinity of tumors [149], and in activated microglia of a simian model of AIDS dementia [150]. The first attempts to localize CB_2_R in the CNS in basal conditions failed because CB_2_R could only be seen in pathological conditions, as described before. Nevertheless, the CB_2_R receptor was not only detected in microglia [104,151] but also in neurons [152,153]. Altogether, the CB_2_R receptor expression increases in activated microglia as a response to certain neuropathological and neuroinflammatory conditions [154]. The activation of CB_2_R in microglia by cannabinoids regulates immune functions in these cells, stimulating microglial proliferation and migration and reducing neurotoxic factors such as TNFα or free radicals [155,156,157,158,159] having lower harmful effects in the microglia at the lesion sites [87].

**Figure 1:**
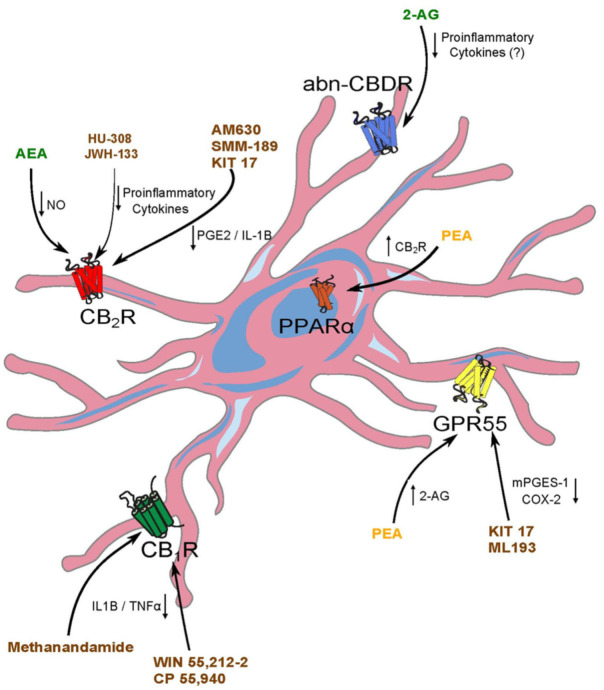
Schematic representation of the interactions of (endo)cannabinoids and synthetic cannabinoid compounds with microglia in the context of epilepsy and neuroinflammation. Endocannabinoids (green) interact with CB_2_ receptors and other non-classical receptors (GPR55, PPAR and abn-CBDR). Fatty acid amides, such as PEA, interact with non-classical receptors (yellow), while synthetic cannabinoid effects (brown) involve mainly CB_1_ and CB_2_ receptors.

Endocannabinoid signaling participates in microglial cell polarization (i.e., different active phenotypes) [99,127,160], potentially affecting the development of neurological disorders. Thus, eCBs drive microglia to the neuroprotective M2 or the resting M0 states [161]. Indeed, M0-microglia is involved in several physiological functions, ranging from plasticity modulation to synaptic pruning and neuronal maintenance [135,162]. In pathological conditions, eCBs and synthetic cannabinoids could dampen nitric oxide (NO) signaling, ROS production, and pro-inflammatory cytokines released from activated M1 microglia [163,164,165]. In line with this, CB_1_R directly affects the inflammatory reaction of microglial cells produced by the immune challenge elicited by the lipopolysaccharide (LPS) model, but not on normal physiological conditions. Furthermore, the CB_1_R genetic deletion attached to CX3-CR1 positive cells results in reduced pro-inflammatory cytokine production and illness amelioration in males, but not in females, pointing out a sexual dimorphism of CB_1_R in these cells [96]. Likewise, the AEA analog methanandamide and synthetic cannabinoids (WIN 55,212-2, CP 55,940) reduce the inflammatory response by suppressing IL-1β and TNFα production in the LPS model of neuroinflammation [166], indicating that CB_1_Rs can mediate changes in inflammatory responses caused by cannabinoids in disease [95] (Figure 1). In addition, microglial CB_2_Rs activation reduces TNFα or free radicals [155,167,168], and AEA-induced NO decrease is mediated by CB_2_R in microglial cells [163] (Figure 1).

In organotypic hippocampal slice cultures treated with L-α-Lysophosphatidylinositol (LPI), a model to study excitotoxicity, activation of GPR55 results on microglial neuroprotection [169]. Interestingly, LPS stimulation has opposite effects on GPR55 expression [163,170]. Furthermore, in LPS-activated microglia, GPR55 antagonists (KIT17, ML193) decrease mPGES-1 and COX-2 without affecting microglial viability [171]. The participation of CB_2_R could not be fully excluded, as the inverse CB_2_R agonist AM630 also decreases PGE_2_ levels in a similar manner, suggesting that both GPR55 and CB_2_ receptors could be mediating the KIT17 effect [171] (Figure 1). In this regard, the CB_2_R selective inverse agonist SMM-189 prevents PGE_2_ and IL-1β increase caused by LPS [172] (Figure 1). Moreover, SMM-189 drives microglial polarization from M1 to M2 state [173,174]. However, the M2 microglial indicator CD206 increases with SR-144528 (a CB_2_R inverse agonist), unlike with the selective CB_2_R agonists HU-308 and JWH-133 [175]. Furthermore, the latter up-regulates the MKP-1 signaling pathway in microglia, lessening microglial activation and reducing pro-inflammatory cytokine release [135] (Figure 1).

AEA also promotes microglial M2 state [176], and the genetic CB_2_R deletion in microglial cells disrupts the polarization to M2 phenotype [127]. A recent review [127] summarizes the current knowledge about the role of cannabinoids in microglial polarization in different pathological situations. Altogether, because of their participation in microglial polarization and function, modulation of microglial CB_2_R and GPR55 could have a direct impact on the development of epileptogenesis after an excitotoxic insult.

#### 4.3.2. Beyond Cannabinoid Receptors: Other Molecular Targets

Apart from the canonical cannabinoid receptors, other microglial-expressed molecular targets have been involved in the neuroprotective effect of cannabimimetic compounds. The neuroprotective effects of the endocannabinoid 2-AG have been described mostly related to CB_1_R or CB_2_R activation in several models of neurological diseases [177,178], and 2-AG, N-arachidonoyl dopamine (NADA), and PEA decrease the number of damaged neurons in hippocampal cultures exposed to excitotoxic insults, probably by reducing microglia [179,180]. However, 2-AG also exerts neuroprotection by a non-canonical cannabinoid-like receptor present in microglia [181]. Furthermore, the abn-CBDR that was characterized in blood vessels [182,183] also participates in glial cell guidance by modulating the release of pro-inflammatory cytokines [118] (Figure 1).

**Figure 2:**
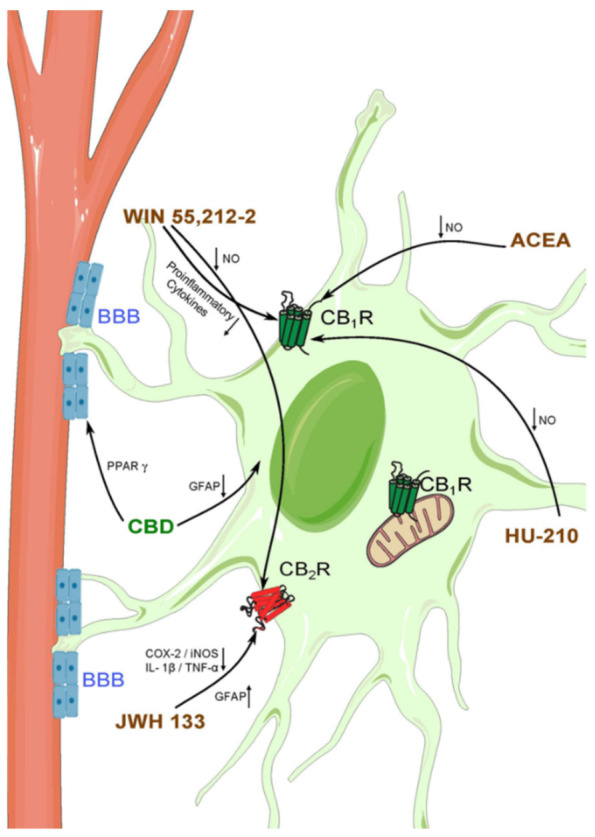
Schematic representation of the interactions of cannabinoid compounds with astrocytes in the context of epilepsy and neuroinflammation. Synthetic cannabinoids (brown) are able to interact with CB_1_ and CB_2_ receptors located in astrocytes. Moreover, some phytocannabinoids (green) modulate astrocyte functions on a cannabinoid-receptor-independent manner.

PEA is a fatty acid amide compound derived from membrane phospholipids produced on demand that exerts anti-inflammatory effects, probably through PPARα receptors expressed in microglia [180,184,185,186,187]. This suggests that cannabinoids acting on microglial PPARα regulate inflammation elicited by excitotoxic insults. Furthermore, PEA increases CB_2_R expression through PPARα [188] (Figure 1) and 2-AG synthesis via GPR55 activation [189] (Figure 1). Therefore, PEA activity in microglia could engage a complex array of cannabinoid and non-cannabinoid molecular effectors [190]. Thus, it seems that besides classical cannabinoid receptors, other cannabinoid-interacting receptors such as the abn-CBDR or PPARα could be a promising therapeutic target for future studies. It is important to note that the molecular targets of endocannabinoids and phyto- or synthetic cannabinoids are not necessarily the same. For instance, in an LPS model, while some effects of cannabidiol (CBD) are through PPARγ, the PPARγ antagonist GW9662 prevents LPS-stimulated microglial activation [191].

## 5. Astrocytes and ECS in Epilepsy

### 5.1. Astrocytes

Until recent years, astrocytes were seen as bare supportive and structural cells from the CNS, providing maintenance to neurons by connecting them with blood vessels. However, nowadays this concept has totally changed and astrocytes entail a prominent role in brain (patho)physiology. Astrocytes present a morphological structure characterized by complex processes that modulate interactions with other neighboring CNS elements, such as neurons, blood–brain barrier endothelial cells, or other glial cells. By doing this, astrocytes modulate synaptic networks regulating physiological processes spanning from metabolism to behavior [192,193,194,195]. For example, astrocytes are crucial for neuronal viability by transporting nutrients from the blood—brain barrier (BBB) in combination with endothelial cells to neurons [196].

Moreover, astrocytes are part of the tripartite synapse [197]. They integrate and process information from the surrounding synapses, mainly due to increased intracellular Ca2^+^ levels and modulation of gliotransmitter release. Thus, they are capable of modulating synaptic plasticity and transmission [198,199]. In particular, astrocytes participate in the clearance of neuronal glutamate from the synaptic cleft, helping in cases of hyperactivation and preventing excitotoxicity [55,200,201]. Therefore, astrocytic malfunctioning could lead to the occurrence of neuronal disorders such as epilepsy [202].

### 5.2. Astrocytes and Neuroinflammation in Epilepsy

In pathological situations such as hypoxic/ischemic insults, excitotoxicity, or demyelinating diseases, specific morphological, molecular, and functional changes occur in astroglia that might be crucial in preserving CNS functioning. The impact of those changes on neurons is far from being homogenous, varying in a context-specific manner from adaptive responses to harmful mechanisms that can aggravate or even cause some neurological disorders [203].

Astroglia has been postulated as a potential target for treating epileptic disorders. In particular, the control wielded by astrocytes in the balance between GABA and glutamate is critical in controlling seizures [204,205]. For instance, impaired glutamate reuptake and adenosine metabolism disruption are linked to epileptiform activity [200,206,207]. Additionally, astrocytes could regulate epileptogenesis by being involved in BBB breakdown and neuroinflammation [208]. Brain inflammation alters gap junction coupling and K^+^ buffering, inducing disturbances that are fundamental in TLE [209]. All the previous changes described might be behind seizure intermittence [208,210].

### 5.3. Astrocytes and ECS in Epilepsy

The role of the ECS in epilepsy has been thoroughly studied. However, the specific participation of astrocytic cannabinoid signaling has not been greatly considered, even though their involvement seems more than plausible to occur [211]. Thus, an augmented expression of CB_1_Rs in astrocytes in the sclerotic rat hippocampus has been reported after three days and four weeks of pilocarpine-induced status epilepticus. Additionally, CB_1_R expression increases in hippocampal astrocytes from patients with refractory TLE and hippocampus sclerosis [212]. In addition, it has been demonstrated that the pharmacological blockade of CB_1_Rs prevents calcium elevations in astrocytes, and reduces hippocampal epileptiform activity induced by 4-aminopyridine (4-AP) [213]. This suggests that endocannabinoid-mediated interactions between neurons and astrocytes are necessary for the maintenance of seizures. Furthermore, the anti-convulsant effect of CBD was linked to GFAP decrease in CA1 and CA3 hippocampus, probably due to the reduction in astrocyte hyperplasia [214]. In co-cultured human astrocytes and brain endothelial cells, CBD reduces BBB permeability in a PPARγ-dependent manner [215] (Figure 2).

The synthetic cannabinoid arachidonyl-2′-chloroethylamide (ACEA) has anti-convulsant properties and shows interactions with various anti-epileptic drugs potentiating their effects in different epilepsy models [216,217,218]. Thus, ACEA-induced potentiation of valproic acid (VPA) increases newborn astrocytes in the hippocampus, suggesting a possible implication of the cannabinoid signaling in neurogenesis after epileptic insults [219]. Furthermore, the activation of CB_1_Rs by ACEA and other synthetic cannabinoids (WIN55 212-2, HU-210) suppresses NO release in LPS-stimulated cultured astrocytes [121] (Figure 2), and WIN55 212-2 through CB_1_R and CB_2_R inhibits NO production and inflammatory molecules in astrocytic cultures [122] (Figure 2).

Epileptiform-like activity triggered by Mg2^+^ free medium in cortical astrocytic cultures, decreases S phase but increases G1 phase astrocytes, suggesting that G1-S phase transition is disrupted during epilepsy [106]. Furthermore, the CB_2_R agonist JWH-133 upregulates S phase astrocytes and increases GFAP expression (Figure 2). Interestingly, the enhanced astrocyte viability was mediated by PI3K-AKT signaling [106], a pathway that is dysregulated in several CNS disorders and epilepsy [220]. The JWH-133 also reduces the pro-inflammatory molecules COX-2, inducible nitric oxide synthase (iNOS), IL- 1β and TNFα produced by 1-methyl-4-phenylpyridinium (MPP) in astrocyte primary cultures [221] (Figure 2). Furthermore, the neuronal damage caused by complement 3 (C3) in astrocytic cultures treated with 4-AP was significantly reduced in the absence of the vanilloid receptor TRPV1, suggesting that TRPV1 in astrocytes participates in the neuronal loss elicited in epilepsy models [117].

Over the last decades, experimental evidence points out a functional connection between neuroinflammation and ROS, and their participation in epileptogenesis [11,222,223]. The release of pro-inflammatory cytokines causes oxidative stress and exacerbates seizure generation [224]. However, both inflammation and ROS increase can be at the same time the cause and consequence of seizures [225]. Unlike neurons, energetic demand of astrocytes relies on glycolysis. Therefore, they produce more ROS that ultimately regulate neuronal survival by modulating glucose and brain redox [226]. Additionally, activation of CB_1_Rs localized in astrocytic mitochondria [84,88] reduces mitochondrial soluble adenyl cyclase (AC) activity and PKA-dependent phosphorylation, decreasing complex I activity and mitochondrial ROS. Ultimately, mitochondrial CB_1_Rs in astrocytes interfere with glucose metabolism and lactate production that affect neuronal functions and behavior [93]. Then, it is more than plausible that the CB_1_R located in astroglial mitochondria raises as a new potential target to treat seizures and reduce epileptogenesis. Further research is needed to elucidate this intriguing possibility.

## 6. Oligodendroglia and ECS in Epilepsy

### 6.1. Oligodendrocytes

Brain oligodendrocytes are less abundant than other glial cells, although their participation in cerebral physiology is essential [227]. Their main function is to produce myelin sheaths in the CNS, and drive remyelination processes after damage or in demyelinating disorders [228]. Nevertheless, these cells carry out other functions that contribute to neuronal functions and subsistence, such as a correct propagation of axon potentials or neurotrophic factor production [229].

The oligodendrocytes responsible for the mature myelin production are generated from the oligodendrocyte progenitor cells (OPC). The cells are distributed throughout certain regions in the CNS, being more abundant in the white matter (WM) [230,231].

### 6.2. Oligodendrocytes and Neuroinflammation

The cognitive impairment observed in epileptic patients has been related to a reduced WM volume and abnormalities in gray matter (GM), as a consequence of the persistent inflammation that affects oligodendrocyte normal functioning and associates with oligondendrogliosis in the WM, even though the myelin content is decreased [232,233]. Due to the high metabolic rate, oligodendrocytes are much more sensitive to oxidative stress [229]. Furthermore, they are highly vulnerable to the neuroinflammation produced by cytokines released by microglia [234]. Oligodendrocytes also participate in the immune response as they can act as antigen-presenting cells, leading to the activation of CD8+ T cells [235]. They can also release inflammatory mediators resulting in microglia activation and express cytokine receptors, promoting microglial recruitment to damage sites [234]. Remarkably, blockade of LPS-stimulated pro-inflammatory molecules decreases oligondendrocytic loss and reduces WM impairment [236].

### 6.3. Oligodendrocytes and ECS in Epilepsy

Oligodendrocytes express both CB_1_ and CB_2_ receptors [237]. The ECS manipulation and cannabimimetic compounds modulate migration, proliferation, differentiation, and survival of OPCs [237,238]. Likewise, cannabinoids regulate differentiation and myelination in mature oligodendrocytes [239]. This pharmacological approach could help seizures management, as promoting these events could restore WM. Furthermore, CBD reduces apoptosis and ROS production in cultured OPCs exposed to LPS or interferon γ (IFNγ), which suggests the involvement of the endoplasmic reticulum stress pathway [240]. Additionally, the CB_2_R selective agonist β-caryophyllene, inhibits LPS-induced cytotoxicity and NO, ROS and TNFα disposal in cultured oligodendrocytes [241].

Despite existing few reports linking oligodendrocyte participation in epilepsy, the role of these glial cells in inflammatory processes could also contribute to understand the complexity of seizures. Moreover, bearing in mind that some cognitive impairment in epileptic patients relates to WM deterioration, the amelioration of these deficits with cannabinoids could also help in epilepsy treatment.

## 7. Conclusions

The subcellular dissection of the ECS is a promising target for managing several diseases, including epileptic-like disorders. Although little is known about the underlying molecular machinery implicated in the effects of cannabimimetic compounds in the control of seizures, their efficacy has been extensively observed in the last decade. Actually, CBD has proven to be efficacious as anti-convulsant in Lennox-Gastaut syndrome and Dravet syndrome [21,242]. Although the participation of neurons is undeniable, the involvement of glial cells seems more than plausible. Despite the fact that neurons are the primary cells affected by seizures due to the hyperexcitation unleashed, glial cells are those implicated in the inflammatory response triggered after status epilepticus, which also affects the progression and extension of the damage. Importantly, these cells also express cannabinoid receptors and are able to release eCBs. Additionally, CBD, other phytocannabinoids, and cannabinoid-based synthetic compounds are currently being investigated and used for treating anxiety [243,244], neurological [69] or psychiatric disorders [245], in addition to many others that do not only affect CNS cells, for instance, cancer, autoimmune, metabolic, or inflammation-related diseases [246,247,248].

Altogether, understanding how cannabinoid compounds act on glial cells might ease to better comprehend the pathophysiology of seizures and the brain itself, leading to a better knowledge of other brain disorders. Furthermore, the glial cannabinoid signaling and related molecular effectors might be regarded as novel targets in the management of seizure-like disorders in the near future.
